# Characterization of interaction and ubiquitination of phosphoenolpyruvate carboxykinase by E3 ligase UBR5

**DOI:** 10.1242/bio.037366

**Published:** 2018-12-15

**Authors:** Qingya Shen, Zhiyu Qiu, Wenping Wu, Jimin Zheng, Zongchao Jia

**Affiliations:** 1 College of Chemistry, Beijing Normal University, Beijing 100875, China; 2 Department of Biochemical and Molecular Sciences, Queen's University, Kingston, Ontario, K7L 3N6, Canada

**Keywords:** PEPCK1, E3 ligase, Ubiquitination, HECT N-lobe, Acetylation

## Abstract

Phosphoenolpyruvate carboxykinase (PEPCK1) is ubiquitinated by E3 ubiquitin ligase UBR5, which was thought to be facilitated by the acetylation of Lys70, Lys71 and Lys594 in PEPCK1. Here, we made a series of UBR5 HECT domain truncation variants and, through pull-down assay, showed that the N-terminal lobe of the UBR5 HECT domain is largely responsible for interacting with PEPCK1. We mutated all three lysine residues thought to be acetylated in PEPCK1 but were surprised to observe no loss of binding to UBR5 HECT domain. Furthermore, two PEPCK1 truncation variants (74-622 aa and 10-560 aa) lacking these lysine residues were still able to bind with UBR5 and ubiquitinated in HEK293T cells. To discover the ubiquitination site(s) of PEPCK1, which is currently unknown, the Lys residues of PEPCK1 were mutated to Ala and the ubiquitination level of the PEPCK1 mutants was assessed. Results revealed at least two ubiquitination sites (Lys243 and Lys342), which represent the first time that ubiquitination sites of PEPCK1 have been identified. Our pull-down experiments further show that the lack of ubiquitination of PEPCK1 Lys243Ala and Lys342Ala mutants is not due to their binding to UBR5, which remained unchanged. Taken together, our work has provided new insights into UBR5 mediated ubiquitination of PEPCK1.

## Abstract (precis)

**Summary:** Identification of the recruit function of the N-terminal lobe of the UBR5 HECT domain and ubiquitination site(s) of PEPCK1.

## INTRODUCTION

Cell metabolism produces many aged and misfolded proteins that need to be degraded all the time. The ubiquitin-proteasome pathway is one of the two protein degradation mechanisms. Compared to the autophagy-lysosome pathway, the protein degradation by the ubiquitin-proteasome system is a more efficient and more specific pathway. In the ubiquitin-proteasome pathway, ubiquitination of protein substrate by E3 ligases is the key step of the three-enzyme cascade (E1, E2 and E3) to enable its protein target specificity. Once ubiquitinated, modified proteins are recognized by proteasome to be degraded. Ubiquitination is the post-translational modification (PTM) of proteins in which the C-terminal glycine residue of ubiquitin and the ε-amino group of lysine residue on substrate proteins form an isopeptide bond (Foot et al., 2017; Pickart, 2001; Sulistio and Heese, 2016). This process requires cascade reactions of three enzymes, including ubiquitin-activating enzyme (E1), ubiquitin-conjugating enzymes (E2) and ubiquitin ligase (E3). E1 activates ubiquitin in an adenosine triphosphate (ATP)-dependent manner and forms a thioester bond through the C-terminus of ubiquitin and the catalytic cysteine residue. Subsequently, the activated ubiquitin is transferred to E2, and then through E3 to substrate proteins. Of the three types of ubiquitin associated enzymes, over 600 E3 ubiquitin ligases in human cells specifically recognize a large number of substrate proteins (Li et al., 2008). Thus, substrate specificity is primarily achieved through E3 enzymes, dictating the temporal and spatial degradation of a variety of proteins in cells.

Based on the ubiquitination mechanism and the domain structure of E3 ligase, they are grouped into three main types (Morreale and Walden, 2016): RING E3 ligase (Deshaies and Joazeiro, 2009), HECT (homologous to the E6AP carboxyl terminal) ligase (Scheffner and Kumar, 2014) and the RBR (RING-Between-RING-RING) E3 ligase (Spratt et al., 2014). Given the importance of E3 ligases, the mechanism by which E3 ligases recognize and ubiquitinate substrates has been a very attractive research topic. It is also very useful in understanding various diseases concerned with protein degradation.

UBR5, also known as EDD, EDD1, HHYD, KIAA0896 or DD5, is an intriguing E3 ligase that belongs to the HECT family (Callaghan et al., 1998). UBR5 is involved in many cellular functions such as DNA damage response (Maddika and Chen, 2009; Zhang et al., 2014), transcription (Su et al., 2011), apoptosis (Eblen et al., 2003) and cell signaling (Hay-Koren et al., 2011) etc. through ubiquitinating its substrate proteins. UBR5 not only acts as a standard HECT family member that recognizes and ubiquitinates substrate proteins by itself, but it also forms an EDVP E3 ligase complex which resembles RING E3 ligase together with DDB1 and VPRBP proteins (Hossain et al., 2017; Jung et al., 2013; Maddika and Chen, 2009). UBR5 has a highly conserved HECT domain at C-terminus. It also contains two nuclear localization sequences (NLS) (Henderson et al., 2002) and three protein interaction domains: an ubiquitin associated (UBA) domain (Kozlov et al., 2007) which locates at N-terminus, a zinc finger ubiquitin recognin box (UBR) domain (Tasaki et al., 2005) which locates at the middle section of UBR5 and a PABC/MMLE domain (Muñoz-Escobar et al., 2015; Yoshida et al., 2006) which is adjacent to the HECT domain. The ubiquitin catalytic HECT domain consists of the N-lobe and C-lobe, which are connected by a flexible linker. Although the C-lobe of the HECT domain of UBR5 contains a conserved cysteine which is responsible for forming a covalent bond with ubiquitin (Matta-Camacho et al., 2012), it does not form non-bonded interactions with ubiquitin and UBCH4 (Honda et al., 2002; Matta-Camacho et al., 2012), which is the upstream E2 enzyme of UBR5. This is very different and unique compared to other HECT family members such as Nedd4 (Kamadurai et al., 2009). Thus, the function of the N-lobe in UBR5 HECT domain is not clear and more in-depth characterizations of UBR5 HECT domain are needed.

Thus far, more than two dozen of proteins were found to be ubiquitinated by UBR5, which are associated with many pathways of cellular processes (Shearer et al., 2015). PEPCK1 (phosphoenolpyruvate carboxykinase 1, also known as PCK1 and PEPCK-C) is one of UBR5's substrate proteins which is involved in gluconeogenesis (Jiang et al., 2011). As the key cellular process for maintaining normal blood sugar in healthy individuals, the gluconeogenesis pathway generates glucose from other non-carbohydrate carbon substrates. PEPCK1 catalyzes the formation of phosphoenolpyruvate (PEP) from oxaloacetate (OAA) along with guanosine triphosphate (GTP), with the release of side products guanosine diphosphate (GDP) and CO_2_. This reaction is the rate-limiting step of gluconeogenesis, thus the level of PEPCK1 in cells plays a most critical role in maintaining the glucose homeostasis (Dunten et al., 2002; Matte et al., 1996). It is therefore not surprising that the PEPCK1 level in cells needs to be carefully regulated, since misregulation can lead to serious conditions including type 2 diabetes. One of the main mechanisms to control PEPCK1 levels is ubiquitination-mediated degradation. In this process, E3 ligase UBR5 is such a specific enzyme involved in PEPCK1 degradation. Additionally, PEPCK1 could also be sumoylated by ubiquitin-conjugating enzyme 9 (Ubc9), which induces PEPCK1 degradation through ubiquitin proteasome system (Bian et al., 2017).

Previous cell biology studies suggested that the acetylation of PEPCK1 seems to enhance its degradation and high level of acetylation of PEPCK1 appears to promote its interaction with UBR5. These results indirectly indicate that the interaction between UBR5 and PEPCK1 is mediated by PEPCK1's acetylation sites, which were identified as Lys70, Lys71 and Lys594 (Jiang et al., 2011). However, the exact domain in UBR5 responsible for the interaction is unknown. Whether binding between the ligase and PEPCK1 is only related to substrate recognition or also acts to affect catalysis is also elusive. Additionally, the ubiquitination site(s) of PEPCK1 is thus far unidentified. In this work, we sought to reveal which UBR5 HECT domain is responsible for interacting with its substrate PEPCK1. We showed that the PEPCK1 lysine residues thought to be acetylated neither played a role in regulating ubiquitination, nor affected binding. By systematically screening PEPCK1 lysine residues, we identified two potential ubiquitination sites for the first time.

## RESULTS AND DISCUSSION

### Cloning, expression and purification of UBR5 HECT truncation variants

**Fig. 1.**
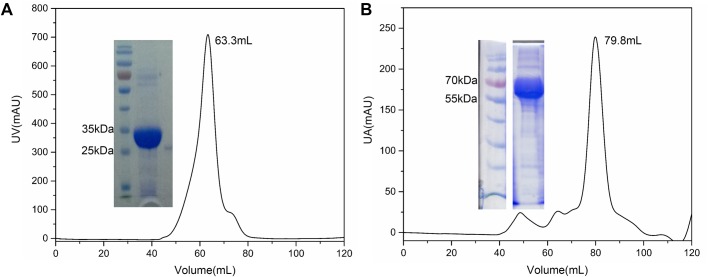
**Purification of UBR5-2-His and UBR5-5-His.** These two proteins were purified by HiLoad 16/600 Superdex 200 pg, in which the column volume was ∼120 ml. Purified protein samples were analyzed by SDS-PAGE. (A) SDS-PAGE shows that UBR5-2-His truncation is of high purity with a molecular weight of ∼31 kDa. The retention volume of UBR5-1 truncation is 63.3 ml, which indicates that UBR5-2 truncation is oligomer. (B) UBR5-5-His is of good purity with little degradation. This truncation acts as a 64 kDa monomer, corresponding to the 79.8 ml retention volume.

UBR5 is a large protein containing 2799 amino acids. Structure prediction of UBR5 shows that it contains significant amount of non-secondary structure, featuring ∼52% random coil (Fig. S1) (Combet et al., 2000). In contrast, the HECT domain of UBR5 has significantly more regular secondary structure than other regions of UBR5. Additionally, UBR5 HECT domain is the catalytic domain with ubiquitin ligase activity and interacts with its substrate PEPCK1. Thus, we focused our work on the HECT domain and constructed a series of UBR5 truncations (Table 1). UBR5-1 to -3 were designed to screen best expression conditions for the HECT domain. UBR5-4 construct contains the PABC and HECT domain of UBR5. UBR5-5 construct was based on another HECT domain crystal structure (PDB ID: 3H1D), which belongs to UBR5's homologous E3 ligase protein HUWE1 (Pandya et al., 2010). UBR5-6, UBR5-7 and UBR5-8 are three variants derived from the UBR5-5 construct. UBR5-9 and UBR5-10 are the N-lobe and C-lobe of the HECT domain, which were designed due to their distinct structure. Most of these constructions expressed well in *E. coli*, except the UBR5-4 construct, which expressed as inclusion bodies. Additionally, UBR5-6 and UBR5-7 were very poorly expressed, making them unamendable for further characterizations. The preparation of UBR5-2 was the best amongst these constructs. We obtained high purity of the HECT domain after nickel affinity chromatography column. UBR5-2 protein formed oligomer when it was purified by size exclusion chromatography (SEC) (Fig. 1A). UBR5-5 is another truncation which also purified well by nickel column, despite a small amount of degradation. In comparison, this truncation was a monomer in solution shown by SEC (Fig. 1B). The constructs were subjected to extensive crystallization trials, but thus far diffraction-quality crystals have not been obtained, despite several promising hits.

**Table 1.**
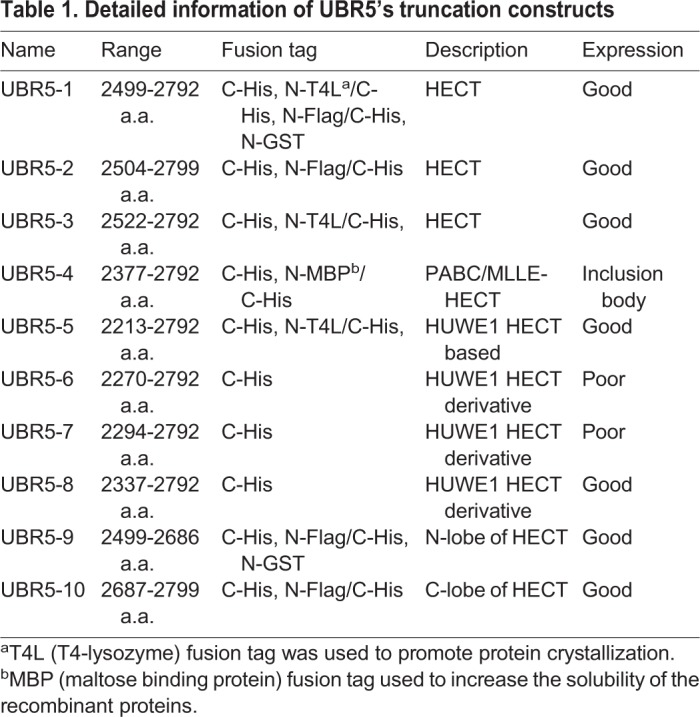
**Detailed information of UBR5's truncation constructs**

### Pull-down assays of HECT truncations with PEPCK1

**Fig. 2.**
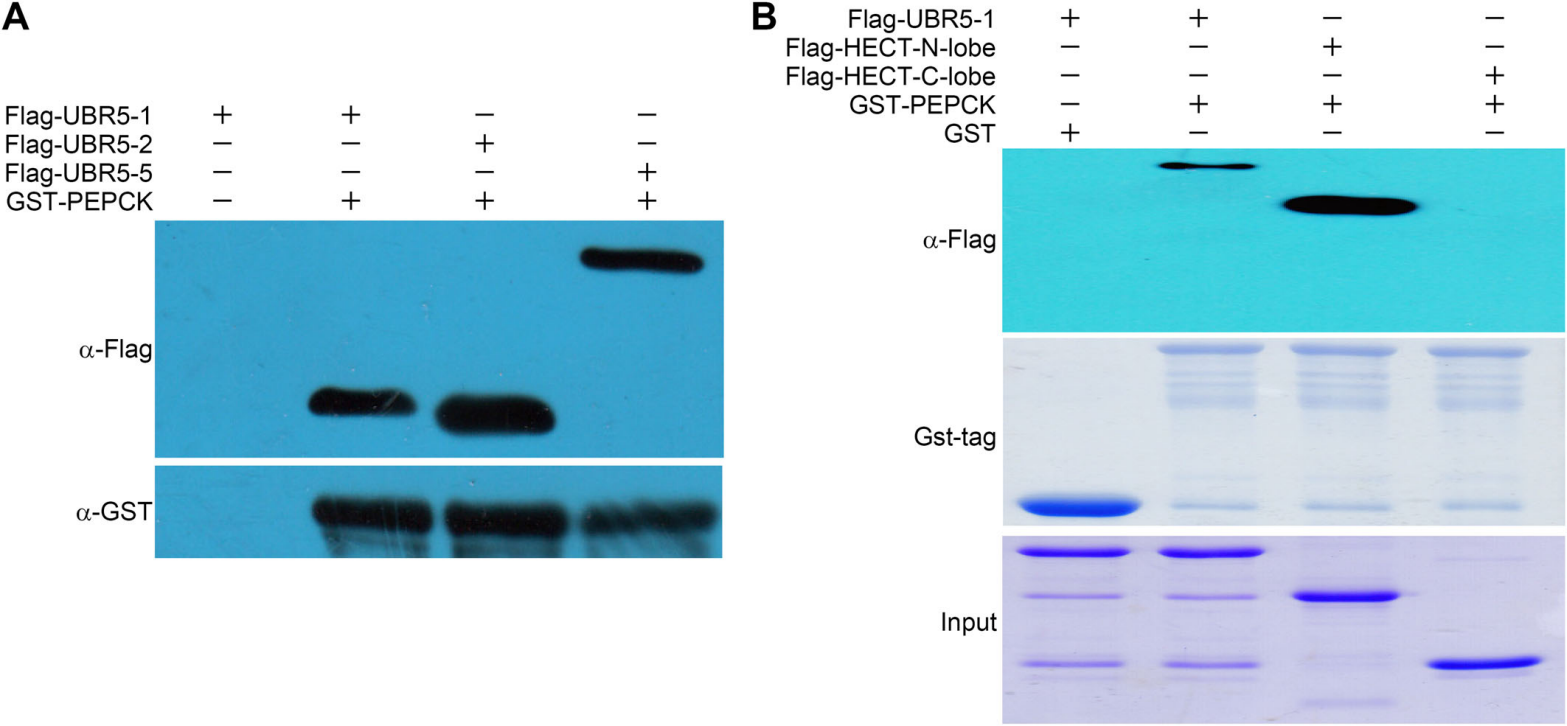
**Pull-down assays of HECT truncations with PEPCK1.** (A) GST-PEPCK pull-down with three UBR5 truncation derivatives. Result shows that the HECT domain is sufficient to interact with PEPCK1. (B) Pull-down assays of HECT N-lobe (Flag-UBR5-9) and HECT C-lobe (Flag-UBR5-10) with GST-PEPCK1. Result shows that the N-lobe has a strong interaction with PEPCK1 while C-lobe does not.

Previous studies found that the C-terminal domain (2375-2799 aa) of UBR5 recognizes and interacts with its substrate (Jiang et al., 2011). In order to characterize UBR5-HECT PEPCK1 binding in more detail, we further cloned three Flag-tagged UBR5 HECT constructs (Flag-UBR5-1, Flag-UBR5-2 and Flag-UBR5-5), along with GST-tagged full-length PEPCK1. These four constructs were successfully expressed in *E. coli* and used in pull-down experiments. The result shows that all three UBR5 HECT constructs bound with PEPCK1 (Fig. 2A), confirming the HECT domain of UBR5 is indeed responsible for recognizing and binding to its substrate PEPCK1. Since the HECT domain is composed of two lobes separated by a flexible linker, we next prepared two additional Flag-tagged constructs of HECT N-lobe (UBR5-9) and HECT C-lobe (UBR5-10). As shown in Fig. 2B, the GST-PEPCK1 fusion protein pulled down the N-lobe truncation but not the C-lobe of HECT domain. Interestingly, the interaction between HECT N-lobe and PEPCK1 seems stronger than the interaction between the full-length HECT domain and PEPCK1. This result indicates that UBR5 recognizes and binds to PEPCK1 through the N-lobe of the HECT domain. UBR5 ubiquitinates PEPCK1 through the C-lobe of the HECT domain, which contains the conservative catalytic cysteine residue.

We have now observed that PEPCK1 interacts with the HECT domain of UBR5 instead of the PABC/MLLE domain, which recognizes another UBR5 substrate, namely polyadenylate-binding protein-interacting protein 2 (PAIP2) (Yoshida et al., 2006). Previous studies of other HECT E3 ligases revealed that the HECT domain is divided into N- and C-lobes (Lorenz, 2018; Ogunjimi et al., 2010). The C-lobe of UBR5's HECT domain possesses the catalytic activity of UBR5 while the function of the N-lobe is not clear. Our results clearly demonstrate that it is the N-lobe of HECT domain that interacts with PEPCK1, and the C-lobe of HECT domain does not bind with PEPCK1. Although more than 20 protein substrates of UBR5 are identified, it is not known which region of UBR5 specifically mediates enzyme-substrate interaction except in the case of PAIP2. Therefore, our work represents the first time that HECT N-lobe has been found to act as a binding domain to recruit substrate. The HECT N-lobe and PABC/MLLE domain both are not far from the catalytic C-lobe of HECT domain, thus it is reasonable that UBR5 recognizes substrate proteins by one of these two domains first to fulfill recruiting function, followed by ubiquitination of substrate through the catalytic C-lobe of HECT domain.

### Lys70, Lys71 and Lys594 of PEPCK1 have little effect on the PEPCK-UBR5 interaction

**Fig. 3.**
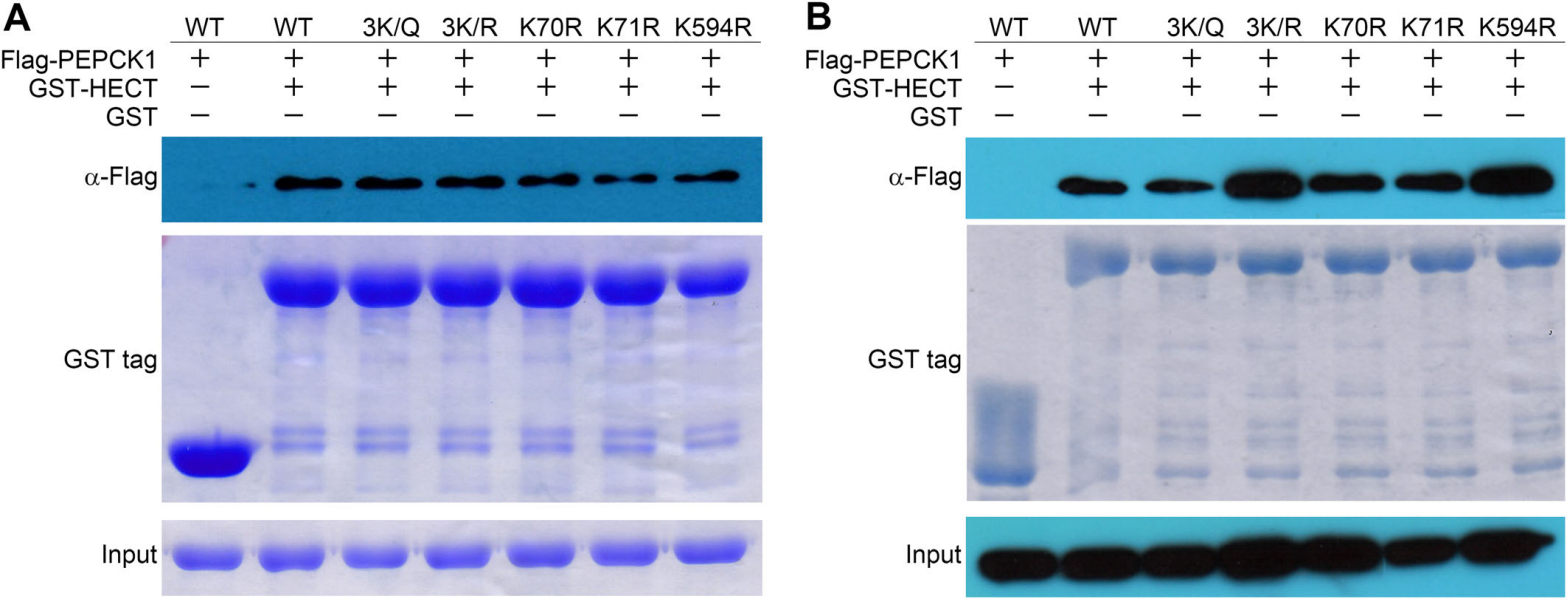
**Pull-down assays of PEPCK1 and its mutants.** (A) GST-HECT pull-down with PEPCK1 and its mutants (3K/Q, 3K/R, K70R, K71R, K594R) *in vitro*. (B) GST-HECT pull-down with PEPCK1 and its mutants (3K/Q, 3K/R, K70R, K71R, K594R) *in vivo*. Results indicate that the mutants of these three acetylation sites of PEPCK1 do not affect its binding with UBR5.

In the ubiquitin-proteasome pathway, the E3 ubiquitin ligase recognizes and ubiquitinates protein substrates, followed by the proteasome-mediated degradation of ubiquitinated proteins. In many cases, ubiquitination is regulated by PTM of the protein substrate. For PEPCK1, it was considered that its ubiquitination level is controlled by acetylation on three sites (K70, K71 and K594) (Jiang et al., 2011). Crystal structure of PEPCK1 reveals that K70 and K71 lie in the flexible loop between two β-sheets, K594 lies in the middle of a rigid α-helix (Fig. S2, PDB ID: 1KHG) (Dunten et al., 2002). The distance between K70/K71 and K594 is very large since they are located at the opposite side of the protein. To investigate this further, we generated acetylation-mimicking mutant (K70Q/K71Q/K594Q), as well as acetylation disabled mutants (K70R, K71R and K594R individually and K70R/K71R/K594R together). As seen in Fig. 3, GST-HECT fusion protein pulled down PEPCK1, the acetylation mimetic PEPCK1^3K/Q^ mutant as well as the PEPCK1^3K/R^ mutant. GST-PEPCK1 also pulled down K70R, K71R and K594R single mutants. Taken together, in contrast to previous literature (Jiang et al., 2011) these proposed acetylation sites appear to have little effect on the interaction between UBR5 HECT domain and PEPCK1.

**Fig. 4.**
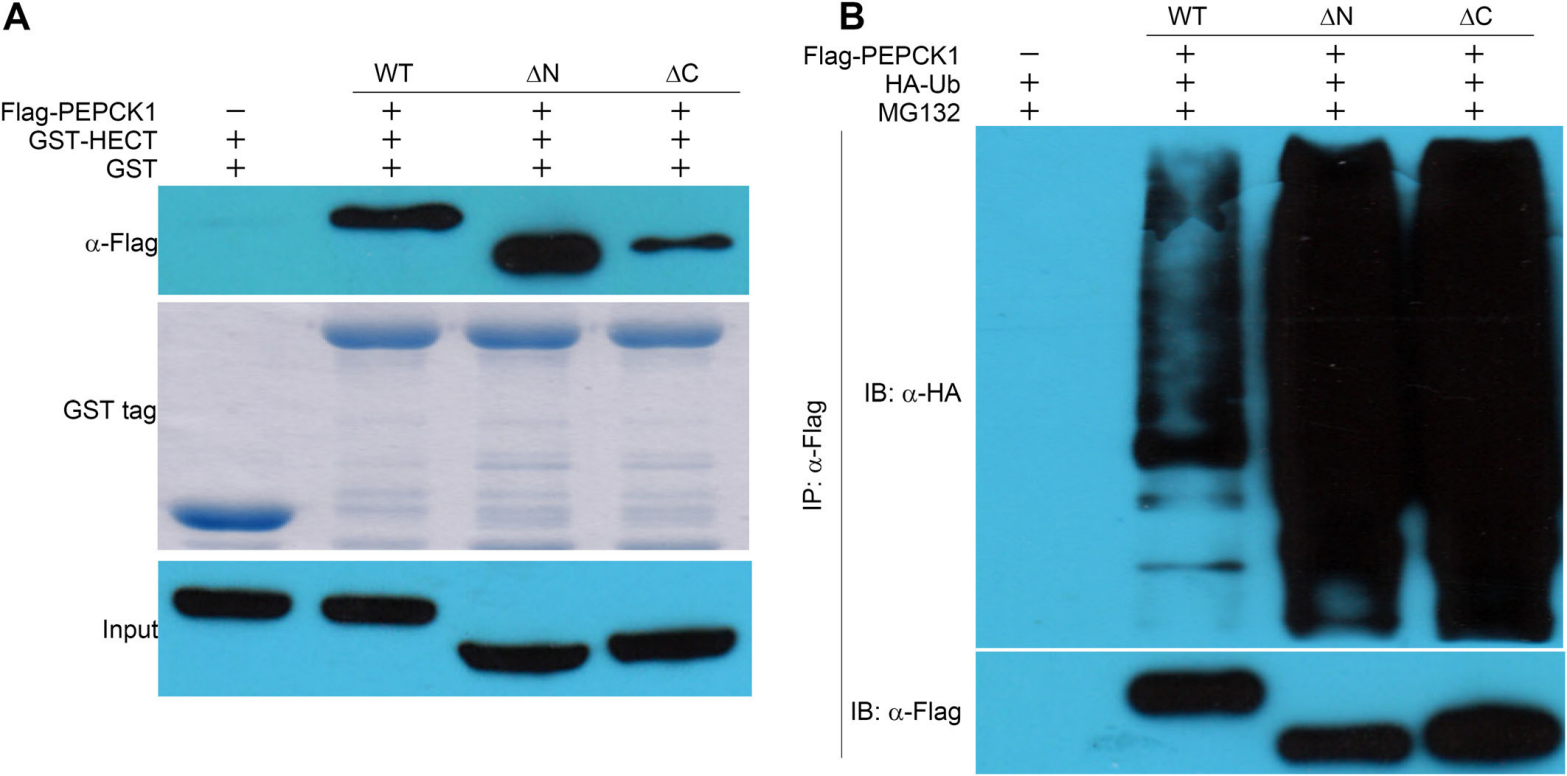
**Pull-down and ubiquitination assays of PEPCK ΔN and ΔC *in vivo*.** (A) GST-HECT pull-down with PEPCK ΔN (74-622 aa) and ΔC (1-560 aa). (B) Ubiquitination assays of these two PEPCK truncations expressed in HEK293T cells. Results indicate that PEPCK1 truncations which feature the loss of acetylation sites still could be recognized and ubiquitinated by UBR5.

These results indicate that K70, K71 and K594 residues may not be responsible for the interaction between E3 ligase UBR5 and its substrate PEPCK1. Additionally, we also constructed ΔN (74-622 aa) and ΔC (1-560 aa) truncations of PEPCK1 which do not contain K70/K71 and K594 respectively and overexpressed them in HEK293T cells. The GST-HECT fusion protein pulled down both truncations (Fig. 4A) and both were ubiquitinated in HEK293T cells (Fig. 4B).

The ubiquitin-proteasome pathway gets rid of aged and misfolded proteins which are recognized and ubiquitinated by E3 ligase. In most cases, PTM (for instance phosphorylation) of substrate proteins is the signal that E3 ubiquitin ligases sense for targeting substrate proteins. Although previous studies indicate that the acetylation of PEPCK1 activates the interaction between PEPCK1 and UBR5 and thus leads to the ubiquitination of PEPCK1, the crystal structure of PEPCK1 clearly shows that the potential acetylation sites (Lys70/Lys71 and Lys594) of PEPCK1 are located as far as possible within the protein (Fig. S2). As such, it would be highly unlikely for all three residues to simultaneously participate in the interaction between PEPCK1 and UBR5. Since we now show that the loss of these three acetylation sites of PEPCK1 (K70, K71 and K594) do not affect the recognition and ubiquitination of PEPCK1 by E3 ligase UBR5, we conclude that these potential acetylation sites are not directly involved in the recognition and interaction of PEPCK1 by UBR5.

The interaction of UBR5 and PEPCK1 may be promoted by other potential acetylation sites of PEPKC1, or other PTMs of PEPCK1 such as phosphorylation. Although many substrates of UBR5 have been discovered, the activation signal to trigger UBR5 reaction is not clear for most of them. In fact, whether acetylation is directly related to ubiquitination remains mysterious. For example, PEPCK1 can be acetylated by P300 which does not help ubiquitination (Jiang et al., 2011). In this particular case, P300 could also enhance acetylation of Ubc9 and hence promotes the simulation of PEPCK1 by Ubc9 (Bian et al., 2017). Sumoylated PEPCK1 is subsequently degraded by ubiquitination which is independent of PEPC1 acetylation. Therefore, our results showing acetylation of PEPCK1 Lys70, Lys71 and Lys594 does not affect ubiquitination by and binding to UBR5 is not surprising.

### Lys243 and Lys342 in PEPCK1 are the ubiquitination sites

**Fig. 5.**
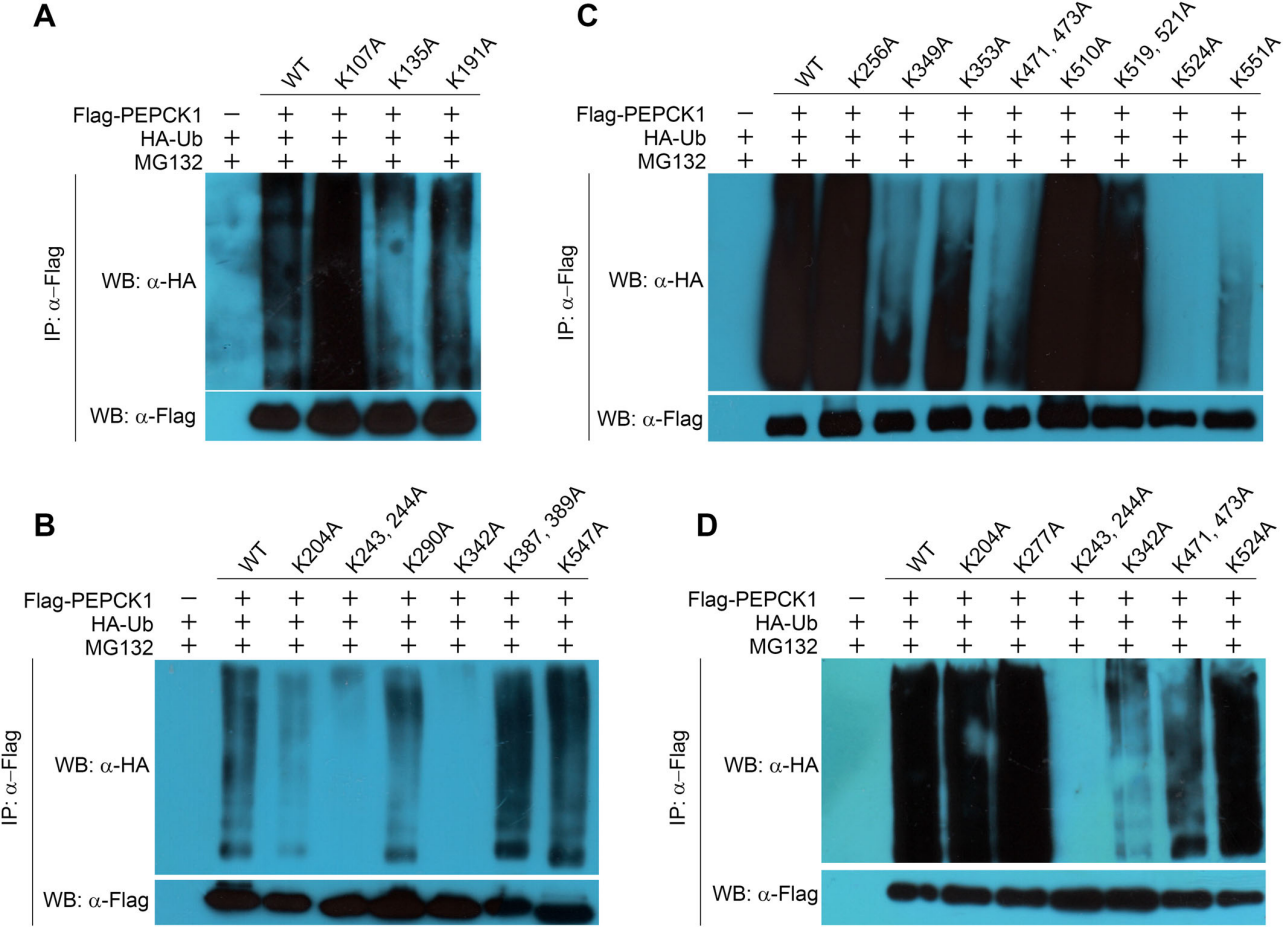
**Screening of ubiquitination sites of PEPCK1.** (A–C) The first round ubiquitination assays of PEPCK1 mutants, 21 Lys sites (107, 135, 191, 204, 243, 244, 256, 290, 342, 349, 353, 387, 389, 471, 473, 510, 519, 521, 524, 547 and 551) of PEPCK1 are screened for the ubiquitination sites. The ubiquitination level of six potential mutants (K204A, K243/244A, K342A, K471/473A, K524A) is much lower than other mutants which need to be validated for another time. Lys243 and Lys342 in PEPCK1 are the ubiquitination sites. (D) The second round of ubiquitination assays of six potential mutants (K204A, K277A, K243/244A, K342A, K471/473A, K524A). The ubiquitination level of K243A, K244A and K342A mutants are much lower than other mutants.

**Fig. 6.**
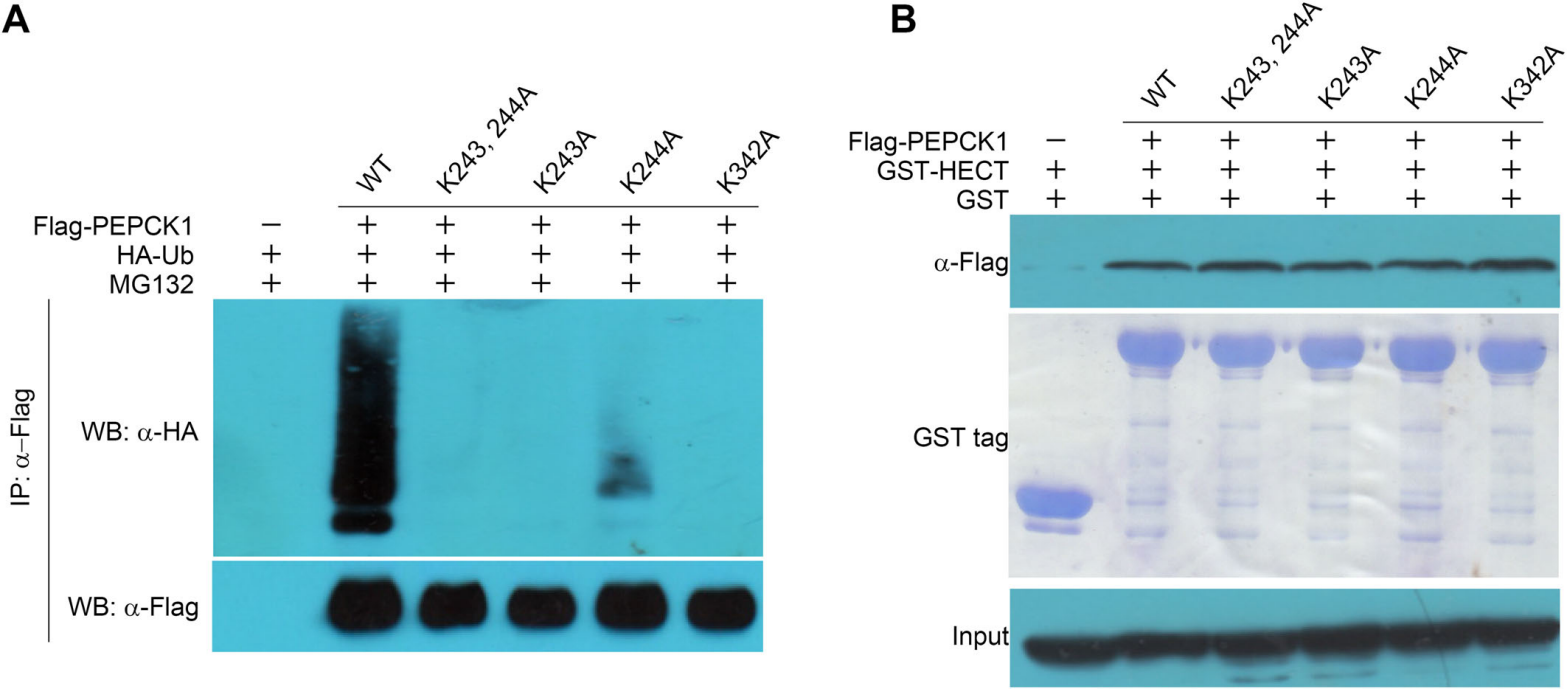
**Lys243 and Lys342 in PEPCK1 are the ubiquitination sites.** (A) Ubiquitination assays of K243A and K342A. K243 and K342 seem to be the two ubiquitination sites of PEPCK1. (B) Pull-downs assays of K243A and K342A mutants. Pull-down assays indicate that these two ubiquitination sites do not affect the interaction between PEPCK1 and UBR5.

PEPCK1 is recognized and ubiquitinated by E3 ligase UBR5, but the ubiquitination site(s) of PEPCK1 remains unknown. Upon overexpressing HA-ubiquitin and Strep-PEPCK1 in HEK293T cells, we purified ubiquitinated Strep-PEPCK1 using Streptavidin affinity resin, followed by SDS-PAGE analysis. The main band containing PEPCK1 was analyzed by liquid chromatography-tandem mass spectrometry (LC-MS/MS) (Fig. S3). Although the acetylation signal was very weak, the MS results indicated that PEPCK1 Lys277 may be a potential ubiquitination site. However, PEPCK K277A mutant was still ubiquitinated when it was co-overexpressed with ubiquitin in HEK293T cells (Fig. 5D). The erroneous MS result may stem from the fact that the abundance of ubiquitinated PEPCK1 peptides was too low to be accurately detected by LC-MS/MS. Next, we decided to analyze the MS results using a process of elimination. Those peptides containing Lys yet detected with high frequency would suggest low ubiquitination probability. On the other hand, there is more uncertainty for the peptides containing Lys but detected with low frequency. We selected and progressively screened 21 Lys sites (107, 135, 191, 204, 243, 244, 256, 290, 342, 349, 353, 387, 389, 471, 473, 510, 519, 521, 524, 547 and 551) by mutating them to Ala based on the MS results listed in Table S2. When co-overexpressing ubiquitin and these PEPCK1 Ala mutants in HEK293T cells, we found the ubiquitination level of five mutants was lower than other mutants in the first round of screening (Fig. 5A–C). In the next round, we selected the five mutants together with K277A mutant and screened their ubiquitination level (Figs 5D and S4). Out of the six mutants, K243A, K244A and K342A seemed to be the most affected, suggesting they are potential ubiquitination sites of PEPCK1. To further confirm these observations, we carried out an additional round of the ubiquitination experiment and the results showed that the ubiquitination of K243 and K342 mutants was barely detectable compared to wild-type PEPCK1 (Figs 6A and S4). These results indicate K243 and K342 residues are likely the ubiquitination sites of PEPCK1. Interestingly, our pull-down results showed that the interaction between the HECT domain of UBR5 and these two PEPCK1 mutants (K243A and K342A) had no difference compared to the wild-type PEPCK1 (Fig. 6B), suggesting that the two residues are largely not responsible for the interaction of UBR5 and PEPCK1.

**Fig. 7.**
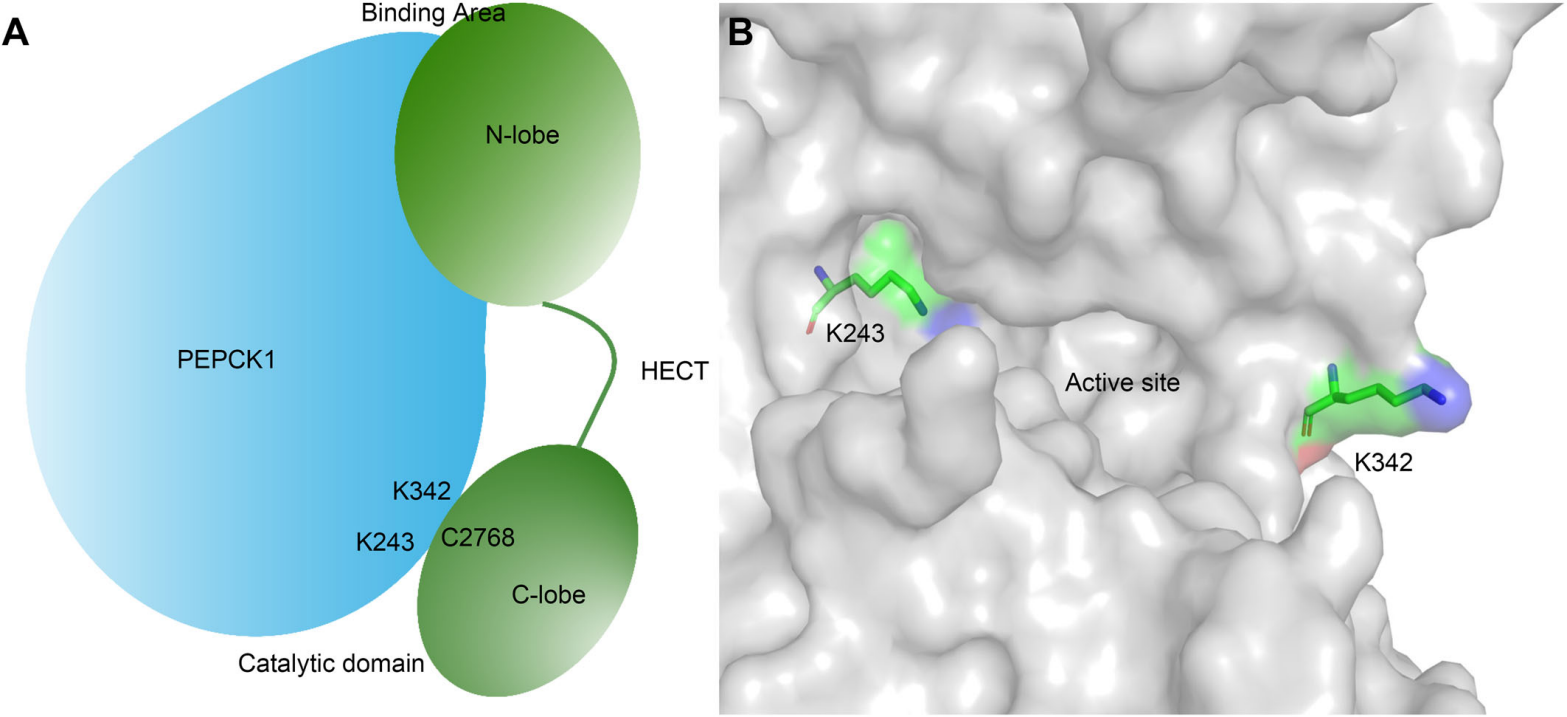
**The role of two ubiquitination sites (K243 and K342) in the interaction between PEPCK1 and UBR5.** (A) Schematic diagram of PEPCK1 and UBR5. UBR5 interacts with PEPCK1 through the HECT N-lobe, and catalyzes the PEPCK1 ubiquitination of K243 or K342 through C2468. The interaction site and catalytic site are of a two-versus-two relationship. (B) Location of the two ubiquitination sites of PEPCK1. The structure of PEPCK1 is shown as surface representation (gray). The two ubiquitination sites (K243 and K342) are shown as sticks and colored by elements. The two lysine residues are positioned at two opposite sides flanking the PEPCK1 active site and exposed to the surface, which makes them amendable to be ubiquitinated by UBR5.

Upon ubiquitination, specific lysine residues of substrate proteins form an isopeptide with ubiquitin enabled by E3 ligase. As we showed earlier, PEPCK1 is recognized or recruited by the N-lobe of HECT domain and ubiquitinated by the C-lobe of HECT domain. As such, the binding area and ubiquitination sites of PEPCK1 are not in the same region. On the substrate side, the binding area and ubiquitination sites also appear to be separated since mutation of the two ubiquitination residues (K243 and K342) does not compromise PEPCK1's binding with UBR5. Taken together, we suggest that two areas of UBR5 HECT (N- and C-lobes) are in contact with the two areas of PEPCK1 (recognition site and ubiquitination site). Although the recognition site of PEPCK1 is not currently known, it would be separated from the ubiquitination site. We have developed a working model to depict such a two-versus-two relationship (Fig. 7A). Both Lys243 and Lys342 are exposed on the surface of PEPCK1 (Fig. 7B), making them accessible for ubiquitination. Interestingly, single mutation of either lysine residue abolishes ubiquitination of the other residue. Since the two lysine residues flank the active site of PEPCK1 (Fig. 7B) relatively closely, it is possible that during the final UBR5-PEPCK1 docking both amino acids are involved to ensure accurate and productive association between the enzyme and substrate. In this case, mutation of one lysine residue may compromise ubiquitination of another.

We cannot rule out an intriguing possibility that ubiquitination of PEPCK1 by UBR5 may serve dual roles – one is to degrade PEPCK1 and another is to directly compromise the kinase activity. The final outcome of both functions would be the same, which prevents phosphorylation of PEPCK1's substrate, OAA. It is known that ubiquitination is not always involved with protein degradation; it can also have functions such as signal transduction. If our proposal that ubiquitination also directly inactivates the kinase is correct, it would be another example of ubiquitination serving a different role distinct from proteasome-mediated degradation.

## MATERIALS AND METHODS

### Cloning of UBR5 and PEPCK1's truncations

The details of UBR5′ HECT domain constructs are listed in Table 1. For protein expression in *E. coli*, constructs were cloned into pGEX-6p-1, pET-22b(+) or pET-28b(+) vector. For protein expression in the mammalian system, constructs were cloned into pcDNA3.0 vector. Clones were constructed by conventional restriction enzyme digestion and ligation. The cDNA of truncations was amplified from human UBR5 gene (GenBank ID: NM_015902) or human PEPCK1 gene (GenBank ID: BC023978) by PCR with desired restriction sites. After purification of PCR products, the PCR fragments and vectors were each digested by the same restriction enzymes. Next, the digested products of PCR fragments and vectors were ligated using Ligation High kit (Toyobo^TM^). The recombinant plasmids were transformed to TOP10 competent cells by heat-shock transformation. After screening clones on agar lysogeny broth (LB) plates and DNA sequencing of the plasmids, the confirmed plasmids were extracted and stored at −40°C.

### Site-directed mutagenesis of PEPCK1

High purity PEPCK1 plasmid was used as template for generating mutants. The mutant PEPCK1 plasmids were amplified by PCR with mutagenic primes (Table S1) using high-fidelity polymerase. If the plasmids were GC-rich, DMSO was added to the PCR reaction (usually around 3% final concentration) to improve the PCR products. Then PCR products were purified and digested by DpnI enzyme at 37°C for 30 min to remove the PEPCK1 template. The digested mutant products were transformed to TOP10 competent cells for screening the desired mutants. Subsequent procedures are much the same as described above.

### Protein expression and purification

For *E. coli* expression, plasmids were transformed to protein expression strains such as BL21 (DE3), BL21 RP, JM109 and BL21 RIL etc. Clones were picked on agar LB plates and seeded into LB culture containing 100 μg ml^−1^ antibiotic (kanamycin or ampicillin). When cells grew to OD_600_=0.8, the culture was induced with 0.5 mM IPTG at 16°C for 24 h. For each construct, various parameters including strains, OD_600_, concentration of IPTG, temperature and cultivation time were varied to screen for the best expression condition.

For His-tagged proteins, cell pellet was suspended in lysis buffer [50 mM Tris-HCl, pH 8.0, 500 mM NaCl, 20 mM imidazole, 10 w/v glycerol, 1 mM PMSF, 0.05% v/v β-mercaptoethanol (β-Me) and 0.1% v/v Triton X-100] and crushed at 700 bar for two cycles. After the cell lysate was centrifuged at 4°C, 20,000 ***g*** for 30 min, the supernatant was allowed to flow through 2 ml Ni-NTA resin (GE Healthcare) which was equilibrated using lysis buffer. The Ni-NTA resin was washed using 100 ml wash buffer (50 mM Tris-HCl, pH 8.0, 500 mM NaCl and 40 mM imidazole), followed by eluting the target protein using 20 ml elution buffer (50 mM Tris-HCl, pH 8.0, 150 mM NaCl and 500 mM imidazole). The protein eluted from the affinity column was concentrated and the buffer was changed to the size exclusion chromatography (SEC) buffer. SEC buffer may be different depending on the target protein. Next, the protein was purified by ÄKTA Purifier system using the SEC column (HiLoad^TM^ 16/600 Superdex^TM^ 200 pg or HiLoad^TM^ 16/600 Superdex^TM^ 75 pg) which was equilibrated using SEC buffer.

For the mammalian expression system, HEK293T cells were cultured in DMEM medium containing 10% v/v fetal bovine serum (FBS) and 1% v/v 100 X penicillin-streptomycin (PS) mixture. About 4 μg plasmids were used for transfecting HEK293T cells which were cultured in a 10 cm Petri dish using linear polyethylenimine hydrochloride (PEI MAX 40K). The concentration PEI stock was 1 mg/ml and the transfection ratio of plasmids and PEI was 1:3 (w/w). Forty-eight hours post transfection, cells were harvested and lysed in 1 ml cell lysis buffer (50 mM Tris, pH 7.5, 150 mM NaCl, 5 mM EDTA, 5% v/v NP-40 and 1 mM PMSF) by vortexing in 4°C for 30 min. The supernatant was to be used for pull-down or other experiments.

### Pull-down experiments

For GST-tagged proteins, cell pellet was suspended in lysis buffer (50 mM Tris-HCl, pH 8.0, 150 mM NaCl, 10% w/v Glycerol, 1 mM PMSF, 0.05% v/v β-Me and 0.1% v/v Triton X-100). The supernatant of bacteria lysates was allowed to bind with GST resin for 3 h. Then the GST resin was washed three times by incubating with lysis buffer for 5 min each. After the GST resin was mixed with another protein lysate overnight, the GST resin was washed three times again by incubating with binding buffer (50 mM Tris-HCl, pH 8.0, 150 mM NaCl) or cell lysis buffer for 15 min each. In the end, the GST resin was resuspended in protein loading buffer and analyzed by western blotting using Flag antibody (BioDee, Cat. #DE0611) to detect the protein–protein interaction.

### *In vivo* ubiquitination assay

Plasmids of Flag-tagged PEPCK1 constructs and HA-tagged ubiquitin were used for co-transfecting HEK293T cells (GE Healthcare, Cat. #HCL4517) at the ratio 1:2. Forty-five hours post transfection, the HEK293T cells were treated with 20 μM MG132 for 3 h. The cells were harvested and treated with the lysis buffer. After the cell lysate was incubated with anti-Flag antibody for 2 h, the lysate was incubated with protein A agarose resin overnight. After discarding the cell lysate, protein A agarose resin was washed three times by incubating with wash buffer (50 mM Tris-HCl, pH 8.0, 250 mM NaCl) for 15 min. Then the resin was resuspended in the protein loading buffer analyzed by western blotting using Flag antibody and HA antibody (CWBIO, Cat. #CW0093M).

## Supplementary Material

Supplementary information
