# Supplementary material for: Characterization of interaction and ubiquitination of phosphoenolpyruvate carboxykinase by E3 ligase UBR5
Source: Biol Open. 2018 Dec 15;7(12):bio037366. doi: 10.1242/bio.037366 (PMC6310884; doi:10.1242/bio.037366)
Supplement: Supplementary information [file biolopen-7-037366-s1.pdf]

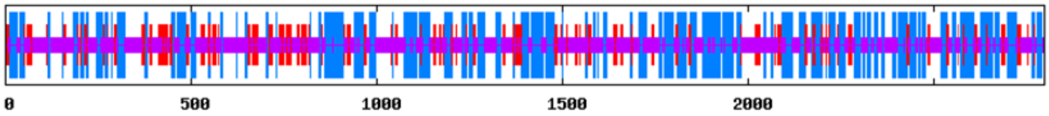

Secondary structure		Number of residues	Percentage
Alpha helix	(Hh)	994	35.51%
3_{10} helix	(Gg)	0	0.00%
Pi helix	(Ii)	0	0.00%
Beta bridge	(Bb)	0	0.00%
Extended strand	(Ee)	360	12.86%
Beta turn	(Tt)	0	0.00%
Bend region	(Ss)	0	0.00%
Random coil	(Cc)	1445	51.63%
Ambiguous states		0	0.00%
Other states		0	0.00%

Fig. S1. The secondary structure prediction of UBR5. Over 50% UBR5's structure is predicted to be random coil. The C-terminus of UBR5 has more α-helix and β-strand structure than other areas of UBR5. The α-helix, β-strand and random coil structure is labeled in blue, red and purple.

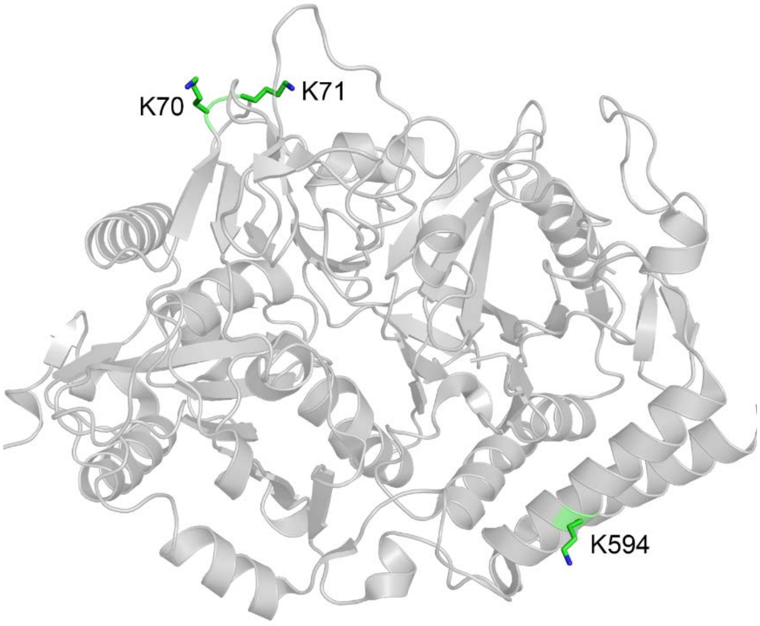

Fig. S2. The location of K70, K71 and K594 in PEPCK1 (**<u>PDB ID: 1KHG</u>**). The overall PEPCK1 structure is shown as cartoon (grey). The three acetylation sites (K70, K71 and K594) are shown as sticks and in green/blue. K70/K71 and K594 are far away from each other.

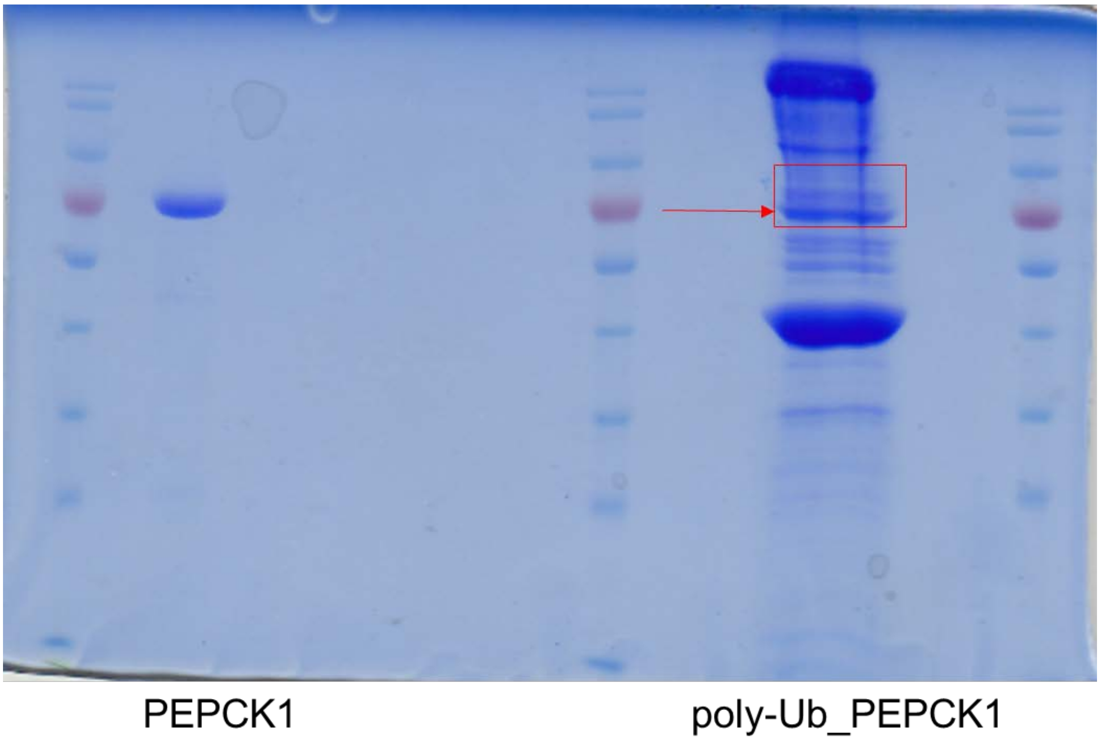

Fig. S3. Purification and LC-MS/MS analysis of poly-Ub_PEPCK1. Purified poly-Ub_PEPCK1 was analyzed by SDS-PAGE. The red rectangle area contains ubiquitinated PEPCK1 which was digested with trypsin and analyzed by LC-MS/MS.Then the digestion was analyzed by liquid chromatography/hybrid linear ion trap-orbitrap mass spectrometry (nanoLC-LTQ-Orbitrap XL, Thermo, San Jose, CA). The data was analyzed using Proteome Discoverer (version 1.4.0.288, Thermo Fischer Scientific) software. The MS2 spectrum was searched using the SEQUEST search engine in the Uniprot_proteome_human_2017 database. During the search process, alkylation of cysteine was set as a fixed modification, while oxidation of methionine and ubiquitination of lysine were set as variable modifications.

Biology Open • Supplementary information

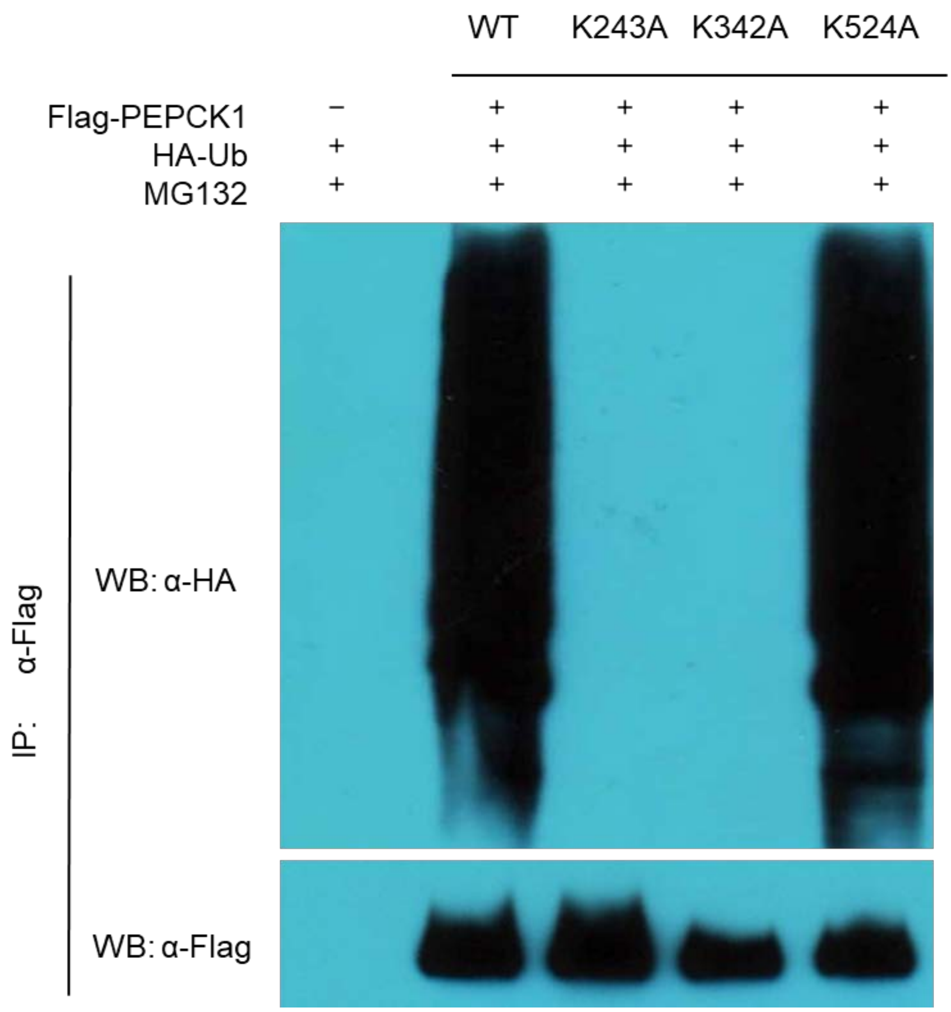

Fig. S4. Ubiquitination assay ruled out K524 as a ubiquitination site of PEPCK1. PEPCK1 WT was used as positive control and the two ubiquitination site mutants (K243A and K342A) were used as negative control.

Biology Open • Supplementary information

Table S1 The mutagenic primers of PEPCK1 mutants

Mutants		Primer sequence
K70,	Forward	5’-GGAAGAGGGCATCCTCAGGCGGCTGCAGCAGTATGACAACTGCT-3’
71Q	Reverse	5’-AGAGCCAACCAGCAGTTGTCATACTGCTGCAGCCGCCTGAGGAT-3’
K70,	Forward	5’-GGAAGAGGGCATCCTCAGGCGGCTGCGGCGGTATGACAACTGCTG-3’
71R	Reverse	5’-GAGAGCCAACCAGCAGTTGTCATACCGCCGCAGCCGCCTGAGGAT-3’
K70R	Forward	5’-GAAGAGGGCATCCTCAGGCGGCTGAGGAAGTATGACAACTG-3’
	Reverse	5’-AGCCAACCAGCAGTTGTCATACCTCTTCAGCCGCCTGAGGA-3’
K71R	Forward	5’-GAGGGCATCCTCAGGCGGCTGAAGAGGTATGACAACTGCTG-3’
	Reverse	5’-GAGAGCCAACCAGCAGTTGTCATACCTCTTCAGCCGCCTGA-3’
K594R	Forward	5’-GAGAAGGAGGTGGAAGACATCGAGAGGTATCTGGAGGATCA-3’
	Reverse	5’-GGCATTGACTTGATCCTCCAGATACCTCTCGATGTCTTCCA-3’
K107A	Forward	5’-GCAAAGAGACACAGTGCCCATCCCCGCAACAGGCCTCAGCCA-3’
	Reverse	5’-GCGACCGAGCTGGCTGAGGCCTGTTGCGGGGATGGGCACTGT-3’
K135A	Forward	5’-CAATGCCAGGTTCCCAGGGTGCATGGCAGGTCGCACCATGTA-3’
	Reverse	5’-TGGGATGACGTACATGGTGCGACCTGCCATGCACCCTGGGAA-3’
K191A	Forward	5’-AGCACTGGGCGATGGGGAGTTTGTCGCATGCCTCCATTCTGT-3’
	Reverse	5’-AGGGCACCCCACAGAATGGAGGCATGCGACAAACTCCCCATC-3’
K204A	Forward	5’-TGTGGGGTGCCCTCTGCCTTTACAAGCGCCTTTGGTCAACAA-3’
	Reverse	5’-GCAGGGCCAGTTGTTGACCAAAGGCGCTTGTAAAGGCAGAGG-3’
K243,	Forward	5’-GTACGGCGGGAACTCGCTGCTCGGGGCGGCGTGCTTTGCTCTCAG-3’
244A	Reverse	5’-GCTGGCCATCCTGAGAGCAAAGCACGCCGCCCCGAGCAGCGAGTT-3’
K243A	Forward	5’-GTACGGCGGGAACTCGCTGCTCGGGGCGAAGTGCTTTGCTCT-3’
	Reverse	5’-GGCCATCCTGAGAGCAAAGCACTTCGCCCCGAGCAGCGAGTT-3’
K244A	Forward	5’-CGGCGGGAACTCGCTGCTCGGGAAGGCGTGCTTTGCTCTCAG-3’
	Reverse	5’-GCTGGCCATCCTGAGAGCAAAGCACGCCTTCCCGAGCAGCGA-3’
K256A	Forward	5’-TCTCAGGATGGCCAGCCGGCTGGCCGCGGAGGAAGGGTGGCT-3’
	Reverse	5’-GTGCTCTGCCAGCCACCCTTCCTCCGCGGCCAGCCGGCTGGC-3’
K277A	Forward	5’-GGGTATAACCAACCCTGAGGGTGAGGCGAAGTACCTGGCGGC-3’
	Reverse	5’-GGGAAATGCGGCCGCCAGGTACTTCGCCTCACCCTCAGGGTT-3’
K290A	Forward	5’-GGCCGCATTTCCCAGCGCCTGCGGGGCGACCAACCTGGCCAT-3’
	Reverse	5’-GGGGTTCATCATGGCCAGGTTGGTCGCCCCGCAGGCGCTGGG-3’
K342A	Forward	5’-CGGTGTCGCTCCTGGGACTTCAGTGGCGACCAACCCCAATGC-3’
	Reverse	5’-GGTCTTGATGGCATTGGGGTTGGTCGCCACTGAAGTCCCAGG-3’
K349A	Forward	5’-AGTGAAGACCAACCCCAATGCCATCGCGACCATCCAGAAGAA-3’
	Reverse	5’-AAAGATTGTGTTCTTCTGGATGGTCGCGATGGCATTGGGGTT-3’
K353A	Forward	5’-CCCCAATGCCATCAAGACCATCCAGGCGAACACAATCTTTAC-3’
	Reverse	5’-GGCCACATTGGTAAAGATTGTGTTCGCCTGGATGGTCTTGAT-3’
K387,	Forward	5’-TTCAGGTGTCACCATCACGTCCTGGGCGAATGCGGAGTGGAGCTCAGA-3’
389A	Reverse	5’-TTCCCCATCCTCTGAGCTCCACTCCGCATTCGCCCAGGACGTGATGGT-3’
K471,	Forward	5’-AGAGGCCACAGCGGCTGCAGAACATGCAGGCGCAATCATCATGCATGA-3’
473A	Reverse	5’-GGCAAAGGGGTCATGCATGATGATTGCGCCTGCATGTTCTGCAGCCGC-3’
K510A	Forward	5’-CCAGCACCCAGCAGCCAAACTGCCCGCGATCTTCCATGTCAA-3’
	Reverse	5’-CCGGAACCAGTTGACATGGAAGATCGCGGGCAGTTTGGCTGC-3’

Biology Open • Supplementary information

K519, 521A	Forward	5’-GATCTTCCATGTCAACTGGTTCCGGGCGGACGCGGAAGGCAAATTCCT-3’
	Reverse	5’-GCCTGGCCAGAGGAATTTGCCTTCCGCGTCCGCCCGGAACCAGTTGAC-3’
K524A	Forward	5’-CTGGTTCCGGAAGGACAAGGAAGGCGCATTCCTCTGGCCAGG-3’
	Reverse	5’-CTCTCCAAAGCCTGGCCAGAGGAATGCGCCTTCCTTGTCCTT-3’
K547A	Forward	5’-GTGGATGTTCAACCGGATCGATGGAGCAGCCAGCACCAAGCT-3’
	Reverse	5’-TATGGGCGTGAGCTTGGTGCTGGCTGCTCCATCGATCCGGTT-3’
K551A	Forward	5’-CCGGATCGATGGAAAAGCCAGCACCGCGCTCACGCCCATAGG-3’
	Reverse	5’-GGGGATGTAGCCTATGGGCGTGAGCGCGGTGCTGGCTTTTCC-3’

Biology Open • Supplementary information

Table S2. The frequency of lysine residues in the peptide fragments detected by LC-MS/MS.

Residues	Frequency	Residues	Frequency	Residues	Frequency
K191	0	K135	8	K243	19
K277	0	K349	8	K560	25
K510	0	K519	8	K389	27
K256	1	K278	9	K107	33
K471	2	K547	9	K316	42
K353	3	K204	11	K124	43
K521	3	K244	13	K155	49
K524	3	K290	17	K492	49
K473	4	K342	17	K304	54
K551	4	K387	17	K507	60

Biology Open • Supplementary information
